# Mediation of the association between social environmental characteristics of family childcare home and weight status in children by diet quality

**DOI:** 10.1186/s12889-023-17179-1

**Published:** 2023-11-21

**Authors:** Qianxia Jiang, Patricia Markham Risica, Alison Tovar, Kristen Cooksey Stowers, Marlene B. Schwartz, Caitlin Lombardi, Kim M. Gans

**Affiliations:** 1 Center for Children’s Healthy Lifestyles and Nutrition, Children’s Mercy Kansas City, Kansas City, MO USA; 2 Department of Behavioral and Social Health Sciences, School of Public Health, Brown University, Providence, RI USA; 3 Center for Health Promotion and Health Equity, School of Public Health, Brown University, Providence, RI USA; 4 Department of Allied Health Sciences, University of Connecticut, Storrs, CT USA; 5 Department of Human Development and Family Sciences, University of Connecticut, Storrs, CT USA; 6 Rudd Center for Food Policy and Health, University of Connecticut, Hartford, CT USA

**Keywords:** Family childcare homes, Diet quality, Childhood obesity, Early childcare

## Abstract

**Background:**

The food and beverages served in family childcare homes (FCCHs) may play an important role in the development of childhood overweight and obesity. This analysis examines whether children’s diet quality mediates the relationship between foods and beverages served in FCCHs and preschool-aged children’s weight status.

**Methods:**

Trained and certified staff conducted observations for two days in each FCCH, using the Environment and Policy Assessment and Observation (EPAO) measure to determine the foods and beverages served to children (*N* = 370) in FCCHs (*N* = 120). They also used the Dietary Observation in Child Care (DOCC) protocol to assess children’s food and beverage intake during childcare, from which we calculated the Healthy Eating Index-2015 (HEI), a measure of diet quality. Height and weight were measured for each child with parent consent from which the child’s body mass index (BMI) z-scores were calculated. A multilevel mediation analysis was conducted to indicate whether children’s diet quality mediates the relations between food and beverage served in FCCHs and preschool-aged children’s weight status.

**Results:**

Children’s total HEI scores significantly mediated the relationship between the EPAO subscale Food Provided and children’s BMI z-scores (B=-0.01, *p* < .05, 95% CI = [-0.03, − 0.002]). Further, the EPAO subscale Food Provided was positively associated with the total HEI score (B = 0.75, *p* < .01, 95% CI = [0.32, 1.18]). Total HEI scores were negatively associated with BMI z-score (B=-0.01, *p* < .05, 95% CI = [-0.02, − 0.001]).

**Conclusion:**

Children’s diet quality did significantly mediate the relationship between the food served in FCCHs and children’s weight status. More longitudinal studies with longer follow-up periods need to be conducted to confirm these relationships. Further, future studies need to examine the relationships between a broader spectrum of FCCH environmental characteristics and home environment with children’s weight status, as well as other mediators including physical activity.

## Introduction

The prevalence of obesity among preschool aged children has increased in the past 30 years [1], with 13.7% of 2-5-year-old children experiencing obesity in 2018 [2]. Childhood obesity is a significant predictor of adulthood obesity [3] and is associated with physical and psychological comorbidities [4–9]. Therefore, it is important to examine how children’s environments, such as childcare settings, may be changed to prevent, instead of contribute to, the risk of excess weight gain [10–13].

Childcare becomes an essential environment for the prevention of childhood obesity given that children who attend childcare spend an average of 35 h per week in such settings [14]. A systematic review in 2018 concluded that the associations between the early childcare social environment and young children’s weight status were likely mediated by the nutrition and physical activity behaviors that affect children’s energy balance [14]. Specifically, the calorically dense foods and beverages served in early childcare settings likely lead to excess energy intake, which in turn affects children’s weight status [14]. Family Childcare home (FCCH) is a type of early childcare setting where a provider cares for a small group of children in his/her home [15]. Compared to center-based early childcare settings, FCCHs have different environments such as having home-based environments, flexible hours, and smaller groups of children at multiple ages [16]. Children who attend out-of-home childcare care, especially less formal types of care such as FCCHs may have an increased risk of childhood overweight or obesity [12, 17–22]. One reason may be that the foods and beverages offered in FCCHs are less likely to meet nutrition standards set by the Child and Adult Care Food Program (CACFP) than in center-based childcare settings [12]. However, to the author’s knowledge, no study has explored whether the food or beverages served in FCCHs is related to children’s weight status mediated through children’s diet quality.

Thus, the aim of the present study is to examine whether the observed food and beverages served in FCCHs are associated with preschool-aged children’s weight status mediated through children’s diet quality. We hypothesized that food and beverage served in FCCH is related to children’s diet quality which in turn is related to children’s weight status.

## Methods

### Participants and FCCHs

The present study utilizes baseline data from Healthy Start/Comienzos Sanos study, an 8-month cluster randomized controlled trial examining the efficacy of a multicomponent intervention to improve nutrition and physical activity environments in English and Spanish-speaking FCCH [23]. Details about study recruitment, intervention, and evaluation as well as study results have been described in full elsewhere, [23, 24] and the methods relevant to the current analyses are described below. The Institutional Review Boards of the University of Connecticut, Brown University, and University of Rhode Island approved all study procedures and materials.

To meet study eligibility requirements, FCCHs had to be within 60 miles of Providence, Rhode Island, and had to have been in operation for at least 6 months. FCCH providers had to read and speak Spanish or English, provide meals and snacks for children, and care for at least two 2-to-5-year-old children for at least 10 h per week. Data was collected from November 2015 to July 2018. Eligible providers completed a baseline telephone survey (13 questions) and in-person survey (26 questions) at the FCCH. Each survey lasts approximately 30 min. A two-day observation was scheduled when at least one parent of an eligible child consented for that child to participate. All measures were conducted or administered by trained project staff. Providers received $25 for completing the baseline in-person survey and $50 for the two-day observation. Children received a reusable water bottle as a thank you gift and parents received a $20 gift card.

### Measures

#### Demographics and other provider  characteristics

Providers were asked to provide information about their sex (male, female, or refuse to answer), race (White, Black or African American, American Indian or Alaska Native, Asian, Native Hawaiian or Other Pacific Islander, other races not mentioned above, unknown), [25] ethnicity (Latinx/non-Latinx) [25] in a telephone survey and the following variables on an in-person survey: age, household income (less than 25k, 25k – 50k, 50k -75k, 75k-100k, more than 100k), marital status (single, married or living with a partner, divorced, separated, widowed), education (less than high school, high school or GED, associate’s degree, Bachelor’s degree, Master’s degree or higher), years in the U.S., country of origin (U.S./non-U.S.), years as a childcare professional, number of children currently in their care (and how many are their own children or grandchildren) and whether the FCCH was enrolled in the CACFP.

#### Weight status

Children’s body weight and height were measured using an established research protocol [26]. The research staff member conducting measurements set up equipment in a space visible to, but located away from the main childcare activities. Children with parental consent who assented to the measurement came to the area to be measured one at a time. Height was measured using a SECA portable stadiometer to the nearest 8th of an inch. Weight was measured using a Tanita digital scale to one decimal place. Measurements were repeated three times and averaged for each child. Body mass index (BMI) was calculated as weight(kg)/height(m²) and BMI z-scores were calculated based on each child’s sex, age and BMI according to the Centers for Disease Control (CDC) growth charts. Per CDC guidance, overweight was defined as BMI > 85th percentile and obesity as BMI > 95th percentile [27].

#### Food and beverage environment in FCCH

To assess the food and beverage environment, we used the validated Environment and Policy Assessment and Observation (EPAO) instrument [28]. The EPAO, originally developed for use in childcare centers, was modified to assess the FCCH environment [29, 30]. One or two field observers (two observers in FCCHs with more than three children) conducted the EPAO observation in each FCCH for two full childcare days, which included at least two eating occasions (breakfast, morning snack, lunch, afternoon snack, and/or dinner). Observations began before children ate breakfast and ended when children left for the day. The nutrition-related sections of the EPAO assess compliance with 38 nutrition best practices. Each practice is rated on a scale of 0–3, where higher scores indicate better compliance. The best practices are grouped and averaged into 7 sub-scale scores, each reflecting an aspect of the overall nutrition environment within FCCHs. In the current study, we used the first two subscales: Food Provided (amount, type, and quality of food provided to children during meals and snacks), and Beverages Provided (amount, type, and quality of beverages provided to children during meals and snacks). The Food Provided subscale included serving of 12 types of food (i.e., whole fruit; fruit with syrup; total vegetables; dark green, orange, yellow vegetables; vegetables with added fat; fried potatoes; fried meat; high-fat meat; low-fat meat; high-fiber whole grain foods; high-sugar high-fat foods; and high-salt high-fat snacks). Beverages Provided subscale included serving 5 beverages (i.e., water; fruit juice; sugary drinks; milk; flavored milk). Observations from the two days were combined to create a single, continuous set of subscores and an overall score. Detailed notes about the FCCH environment and providers’ nutrition and physical activity practices were recorded by the observer during the home visit. Forms were reviewed for accuracy and completeness by field staff. Additional review was conducted by data staff [23].

#### Children’s diet

Children’s dietary quality was measured by calculating the 2015 Healthy Eating Index (HEI) score [31] with two days of dietary data collected using the Dietary Observation in Child Care (DOCC). The DOCC is a reliable, valid visual observation technique for measuring children’s dietary intake developed by Ward and her team [32, 33]. During the DOCC certification process, field staff need to accurately estimate at least 80% of 20 measured portions of food that a child would typically eat. Field staff must achieve 80% inter-rater reliability with a “gold standard” observer in the field at a FCCH to pass the certification. The quality of observations was continually assessed throughout dietary data collection such that observers needed to pass the certification process annually, and participate in structured monthly practice, quarterly validity checks, and semi-annual inter-rater reliability checks. Trained and certified data collectors observed all meals/snacks over two days and estimated the amount of food and beverages served and consumed for each child. These data were entered into Nutrition Data System for Research (NDSR) [34] to calculate an average daily HEI score. The total HEI score is a sum of 13 dietary components sub-scores based on two-day averaged score, ranging from 0 to 100, with higher scores indicating better diet quality [31]. A score of 80 or higher reflects a high-quality diet among preschool aged children [35]. HEI component scores are calculated as intake per 1000 calories (except for fatty acids which is scored as a ratio of unsaturated to saturated fatty acids) with maximum score of 5 or 10 for each component. These components include total vegetables (5), greens/beans (5), total fruit (5), whole fruit (5), whole grains (10), dairy (10), total proteins (5), seafood plant protein (5), fatty acids (10), sodium (10), refined grains (10), added sugars (10), and saturated fats (10) [31].

### Analysis

**Fig. 1:**
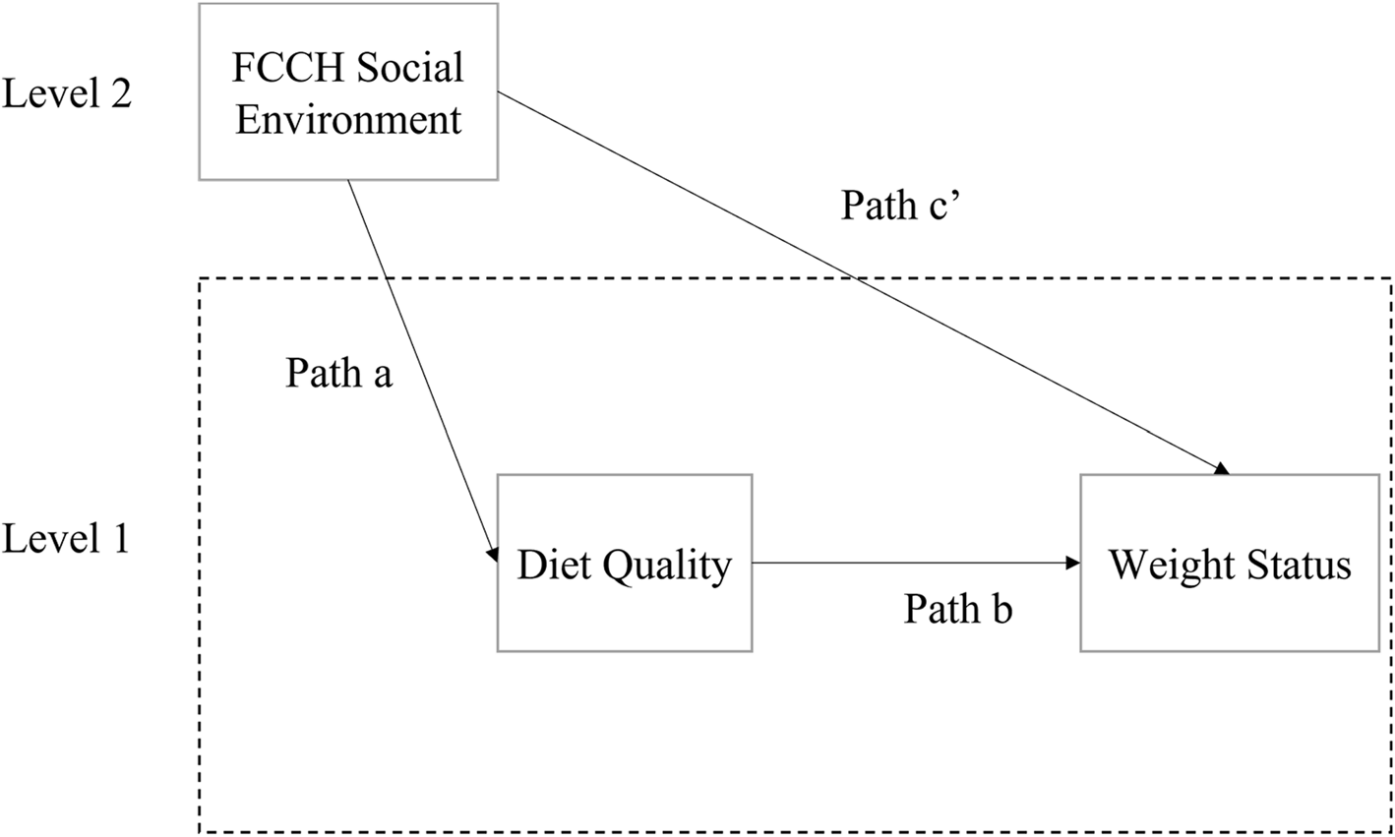
Illustration of a 2-1-1 multilevel mediation model.  Path a represents the effect of the FCCH food environment on child diet quality. Path b represents the effect of child diet quality on child weight status controlling for the FCCH environment characteristics. Path c’ represents the direct effect of Food Provided and Beverages Provided in FCCH on child weight status. All paths represent between-group effects

A multilevel mediation analysis was conducted to examine two questions. First, are there any direct effects of the food and Beverages Provided in FCCHs (measured by the EPAO) on children’s BMI z scores? Second, are these effects mediated by children’s diet quality, as measured by HEI scores? Building on the classic mediation model that assumes independent observations, [36] many mediation analyses have been extended to the multilevel context [37–39]. Many intervention programs are conducted in group settings such as schools and community groups [40, 41]. As individuals within a cluster tend to be more similar than those selected from different clusters, the statistical assumption of independence of units is violated. Thus, statistical multilevel analyses are needed to account for clustering [42] or it may lead to invalid results [43]. The data collection of the current study occurs at two levels. Because multiple children were sampled within each FCCH, all models account for children clustered within FCCHs. In each model, data collected at the child level are called level-1 data, while data collected at the FCCH level are called level-2 data. In the current study, a 2-1-1 model was employed, which corresponds to measurement levels of the independent (i.e., FCCH Food Provided / Beverages Provided – level 2 data), mediator (i.e., child diet quality (HEI) – level 1 data) and outcome (i.e., weight status – level 1 data) variables, respectively (Fig. 1). This two-level model, which allows for grouping of child outcome within FCCHs included residuals at the child and FCCH level [43].

Parameters for the models, including fixed effects of independent variables and mediators as well as random effects of mediators (only random intercepts between mediators and the outcome were estimated), were estimated using restricted maximum likelihood estimation [44]. The FCCH level independent variables did not vary at the individual level; therefore, the within-group effects of the independent variables were omitted and the mediation effects are presented as between-group indirect effects (i.e., the effect of the group differences in independent variables on the outcome through the mediator) [44]. The significance level was set at *p* < .05. A bootstrapping approach was used to determine if this mediation effect is statistically significant [45]. All analyses were conducted in Stata SE 16 [46].

## Results

### Participants

A total of 120 female FCCH providers (67.5% Latinx, 42.5% White, 15% Black, 75% married or living with a partner) were included in the current study. Participant providers averaged 48.9 (SD = 9.0) years old and about 60.8% had yearly household income less than $50K; 43.3% had a high school degree/GED or less. The majority (82.5%) accepted Child and Adult Care Food Program (CACFP) subsidies. See Table 1.

**Table 1:** Family childcare provider demographics

Variable	Category	ALL (*n* = 120) % (n)/ Mean (SD)
Gender		
	Female	100 (120)
Ethnicity		
	Latinx	67.5 (81)
	Non-Latinx	32.5 (39)
Race		
	White/ Caucasian	42.5 (51)
	Black	15 (18)
	American Indiana	3.3 (4)
	Native Hawaii	2.5 (3)
	Other	23.3 (28)
	Multi race	2.5 (3)
	Unknown	10.8 (13)
Country born in		
	US	29.2 (35)
	Non-US	70.8 (85)
Marital status		
	Single	9.2 (11)
	Married or living with a partner	75 (90)
	Divorced	8.3 (10)
	Separated	4.2 (5)
	Widowed	3.3 (4)
Yearly household Income ($)		
	Less than 25k	13.3 (16)
	25k-50k	47.5 (57)
	50k-75kB	20 (24)
	75k-100k	10 (12)
	More than 100k	5.8 (7)
Highest level of education		
	Less than High school	10.8 (13)
	High school or GED	32.5 (39)
	Associates degree	38.3 (46)
	Bachelor’s degree	15 (18)
	Master’s degree or higher	3.3 (4)
Age		48.9 (9.0)
Accept CACFP subsidies		82.5 (99)
Hours work per week as a provider		62.4 (13.8)
Number of Children in the care (include own children or grandchildren)		7.7 (3.1)
Years working in early childcare		12.8 (8.4)
Count of best practices met by providers		11.1 (2.3)
EPAO Food Provided score		2.1 (0.4)
EPAO Beverages Provided score		1.3 (0.5)

A total of 370 children (51% girls, 58% Latinx, 47% White, 10% Black) were included in the current study. Children averaged 3.5 (0.98) years of age. Among children, the mean (SD) BMI z-score was 0.7 (SD = 1.2), and about 35% of children having overweight or obese. The majority of children ate breakfast (84%) and lunch (97%) at the FCCH. On average, they spent 7.6 h per day at the FCCH. See Table 2.

**Table 2:** Child demographics

Variable	Category	ALL (*n* = 370) % (n)/ Mean (SD)
Gender		
	Male	48.6 (180)
	Female	51.4 (190)
Ethnicity		
	Latinx	57.6 (208)
	Non-Latinx	42.4 (153)
Race		
	White/ Caucasian	46.8 (168)
	Black	10.3 (37)
	American Indiana	0.8 (3)
	Native Hawaii	0.8 (3)
	Asian	0.8 (3)
	Other	30.1 (108)
	Multi Race	10.3 (37)
Weight Status (BMI z-score)	Mean (SD)	0.7 (1.2)
	Not Overweight/Obese	65.1 (213)
	Overweight/Obese	34.9 (114)
Child Eats Breakfast at FCCH		83.8 (310)
Child Eats Lunch at FCCH		96.8 (358)
Child Eats Dinner at FCCH		8.4 (31)
Age		3.5 (0.98)
Hours per Day at FCCH		7.6 (0.86)
Total HEI score		62.1 (12.5)

### Multilevel mediation models of diet quality (total HEI scores) in relationships between environment and weight status (zBMI)

The EPAO Food Provided score averaged 2.1 (SD = 0.4) out of 3 and Beverages Provided score averaged 1.3 (SD = 0.5) out of 3 among FCCHs. The child HEI score averaged 62.1 (SD = 12.5) out of 100.

#### Path c

The multilevel mediation analyses did not detect significant associations between the environmental scores (i.e., Food Provided (B = 0.005, p > .05, 95%CI=[-0.03,0.04]) and Beverages Provided (B = 0.013, p > .05, 95%CI=[-0.04,0.07])) and children’s BMI z-scores. Baron and Kenny suggest that if there is no relationship between independent variable and outcome variable, there is no need to test for mediation, however this interpretation is controversial [36]. We moved forward with the mediational analysis because we anticipated that the effect size would be small and we suspected possible suppression [45].

#### Path a

**Fig. 2:**
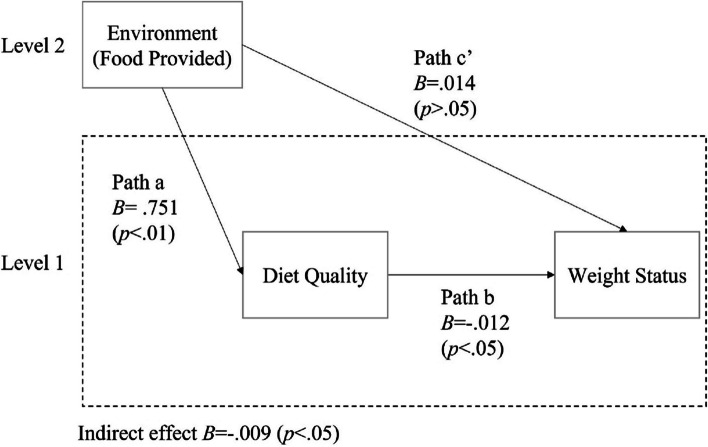
Multilevel mediation models of diet quality in relationships between Food Provided and Weight Status

**Fig. 3:**
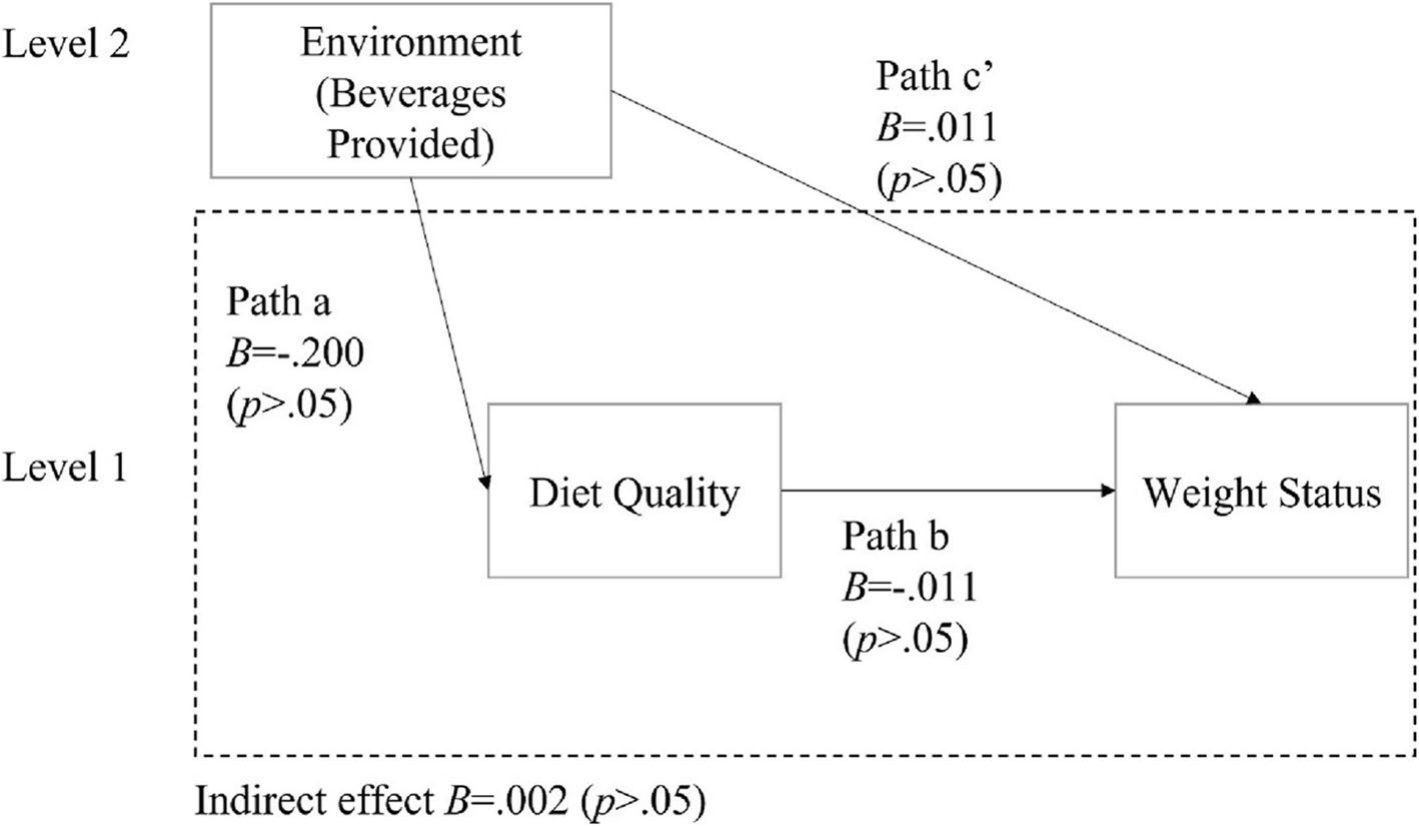
Multilevel mediation models of diet quality in relationships between Beverages Provided and weight status

The environmental subscale Food Provided was positively associated with the total HEI score (B = 0.751, *p* < .01, 95% CI = [0.32, 1.18]). This indicates that a higher score on the Food Provided subscale was associated with better children’s diet quality (Fig. 2; Table 3). However, the environmental subscale Beverages Provided was not significantly associated with the total HEI score, so no further analysis was conducted with beverage variables (See Fig. 3; Table 3).

**Table 3:** Multilevel mediation models of diet quality in associations between FCCHs environment and BMI z-scores

Independent variables	Path a		Path b		Path c’ (Direct Effect)		Indirect Effect		
	B	95% CI	B	95% CI	B	95% CI	B	95% CI	
Food Provided	0.751**	[0.32,1.18]	− 0.012*	[-0.02, − 0.001]	0.014	[-0.02, 0.05]	− 0.009*	[-0.03, − 0.002]	
Beverages Provided	− 0.200	[-0.98,0.58]	− 0.011	[-0.02,0.0001]	0.011	[-0.04, 0.06]	0.002	[-0.01, 0.01]	

*Abbreviation*: *CI *Confidence interval

Note: Path a represents the effect of environment (i.e., Food Provided, and Beverages Provided) on diet quality. Path b represents the effect of diet quality on z-BMI controlling for environment. Path c’ represents the direct effect of environment on z-BMI. The confidence intervals of the indirect effects were estimated using bootstrapping.  All paths represent between-group effects

^*^p<0.05, ^**^p<0.01, ^***^p<0.001

#### Path b and c’

The total HEI score was negatively associated with BMI z-score (B =-0.012, *p* < .05, 95% CI = [-0.02, − 0.001]) when controlling for Food Provided in FCCHs (path b). This suggests that better children’s diet quality was associated with lower weight status when controlling for Food Provided in FCCHs. However, when diet quality was included in the model, there was no significant direct effect of Food Provided on BMI z-scores (path c’), suggesting the effect of Food Provided on BMI z-scores is mediated by children’s diet quality (Fig. 2; Table 3).

There was a significant negative indirect effect of Food Provided scores on BMI z-scores through total HEI scores (B =-0.009, *p* < .05, 95% CI = [-0.03, − 0.002]). There was no significant indirect effect of Beverages Provided on BMI z-scores through total HEI scores (Table 3; Figs. 2 and 3).

## Discussion

The goal of this study was to examine whether preschool-aged children’s diet quality mediates the relationship between FCCH food and beverage environments and children’s weight status, using objective measures of FCCH environments (i.e., food and beverages provided) and children’s diet quality. The study found there is room for improvement in the overall quality of food and beverage served, as well as children’s diet quality. These findings were similar to what others have found about the quality of food/beverage served [10] and children’s diet quality in FCCHs [10, 47]. Further, we found over a third (35%) of the children in the current study were classified in the overweight or obese categories, [48] which is higher than national estimates of 26% overweight/obese prevalence in this age group [2]. Thus, it is urgent to improve the quality of foods and beverages served in FCCHs to promote children’s healthy eating, as well as their healthy weight status. We found children’s total HEI scores significantly mediated the relationship between the EPAO subscale Food Provided and children’s BMI z-scores.

Though the majority (82.5%) of FCCHs in the current study enrolled in CACFP, many providers did not serve enough vegetables and whole grains, make drinking water available and prompt children to drink it, or limit salty/sugary/fatty snacks [13]. Other studies in FCCHs also found that many providers did not implement best practices in serving whole grains and vegetables, and foods that are lower in fat and sugar [12]. However, most of the baseline data collection for the current study was done before October 2017 changes to the CACFP guidelines. The updated guidelines have addressed some of these issues, such as the requirement to offer at least 1 serving of whole grain-rich foods daily and the prohibition of homes from offering fried or pre-fried food on-site [49]. These changes might help encourage children to consume more whole grains and reduce their consumption of high-fat foods in FCCHs.

In mediation models, we found that a higher mean FCCH food-provided score was associated with higher child diet quality in our sample of FCCHs. Further, we found that the FCCH food environment (foods served) in FCCHs indirectly affected child weight status through child diet quality. Similar to previous studies assessing the association between the EPAO Food Provided sub-scale and child diet quality in FCCHs [10, 47, 50], we also found that better FCCH food environments were positively associated with child overall HEI scores. Together, these studies point out the value of improving the quality of the food served in FCCHs in order to promote children’s healthy eating. Consistent with the findings from Benjamin-Neelon et al. (2018), [10] we did not find a significant association between the beverage served EPAO score and child diet quality. One possible reason might be that most FCCHs in the current study served healthier beverages and very few of them served sugar sweetened beverages or excessive juice, so very little variability was assessed in these models. For example, in a prior study conducted with the same sample, it was observed that over 95% of providers consistently offered 100% fruit juice without any added sugar, while refraining from serving sugary drinks [51]. Future studies might identify the associations of Beverages Provided subcomponent scores with the HEI score.

For young children who cannot make health-related choices for themselves, an obesogenic environment is likely to influence young children’s weight status [52, 53] though in the current study, we did not find a significant direct effect of Food Provided in FCCHs on child weight status. Similarly, previous research conducted in center-based childcare settings found that the overall nutrition environment was not significantly associated with preschooler weight status [54]. However, this study found that certain aspects of a healthier food environment such as a lower opportunity for high sugar and high fat foods were associated with lower BMI percentile in preschool-aged children [54]. According to Hayes (2017), mediation without evidence of a total effect of the independent variable means only that on the aggregate, when all paths of influence between independent variable and outcome variable are added up, they are not linearly related [44]. It would be helpful for future studies to examine the associations between obesogenic environment and children’s weight status.

Although previous research conducted in center-based early childcare settings has shown that some aspects of the food environment may affect the development of childhood overweight and obesity, [54] the indirect relationships between social environmental characteristics of early childcare settings and children’s weight status have not been examined. In the current study, we found that the food-served environment in FCCHs indirectly affected child weight status through child diet quality, suggesting that interventions focusing on helping providers to increase healthy foods and decrease unhealthy foods served in FCCHs may play an important role in reducing the risk of childhood overweight and obesity through the improvement of child diet quality. However, the direct and indirect relationships between FCCHs social environmental characteristics and children’s weight status need to be further assessed through longitudinal studies. In addition, other FCCH environmental characteristics that were not included could influence weight status such as provider feeding practices, portion sizes, [13] exposure to opportunities to be physically active, and screen time. Individual-level child characteristics such as temperament, appetite regulation and child preferences, which were not included, may also be associated with weight status.

The current study is the first to examine the associations between food environmental characteristics of early childcare settings and children’s weight status mediating through children’s diet quality, in preschool-aged children. Our use of the observational measures allowed the objective assessment of both the quality of the FCCH food environment and the diet quality of children, which can be more accurate than the subjective self-reported measures used in previous studies. The use of multilevel analysis also allowed us to account for the clustered nature of our data. However, this study does have some limitations. First, the causality of the relationships in the current study cannot be inferred from the cross-sectional design. Additionally, reverse causality is a potential factor to consider. It is plausible that parents who prioritize nutrition may choose FCCHs with better food environments, potentially influencing the associations observed in our study. Future analysis using longitudinal datasets is needed to explore whether changes in FCCH environments lead to changes in children’s weight status over time. Secondly, our sample consisted of a majority of Latinx providers in certain geographic locations, so the results may not be generalizable to a broader population of family childcare providers. Furthermore, only two-day observation data may not be representative of the usual food and beverages served more generally in FCCHs. Future studies could aim to capture the variability in both the types of food and beverages served in FCCH over a more extended period. Additionally, the truncation of the EPAO scores within the range of 0–3 may not fully capture the entire spectrum of variability in the types of food and beverages served in FCCHs. Expanding the score range would likely result in a more complex and impractical assessment process. In the current study, we focused solely on the scores related to food and beverages served, but it is important to acknowledge that other EPAO scores such as feeding practices, may also influence children’s diet quality and weight status. In addition, we did not measure children’s food intake at home, and children’s weight could certainly be affected factors beyond childcare settings. Given that children spend their time both at home and in FCCHs, future studies could explore the cumulative impact of both food and home environments on weight status.

## Conclusion

Overall, the food served in FCCHs has indirect effects on preschool-aged children’s weight status through children’s diet quality, although longitudinal studies need to confirm these relationships. It is crucial to prioritize the implementation of regulations and policies aimed at improving access to healthy foods within FCCHs. Ensuring that appropriate measures are in place will promote healthier dietary choices and prevent childhood obesity.

## Data Availability

The datasets used and/or analyzed during the current study are available from the corresponding author on reasonable request.

## Abbreviations

FCCH: Family Childcare Home
EPAO: Environment and Policy Assessment and Observation
DOCC: Dietary Observation in Child Care
HEI: Healthy Eating Index
BMI: Body Mass Index
NDSR: Nutrition Data System for Research
CACFP: Child and Adult Care Food Program
